# Supplementary material for: Bottom-up precise synthesis of stable platinum dimers on graphene
Source: Nat Commun. 2017 Oct 20;8:1070. doi: 10.1038/s41467-017-01259-z (PMC5715161; doi:10.1038/s41467-017-01259-z)
Supplement: Supplementary file 2 — Supplementary Information [file 41467_2017_1259_MOESM2_ESM.pdf]

Supplementary Figures

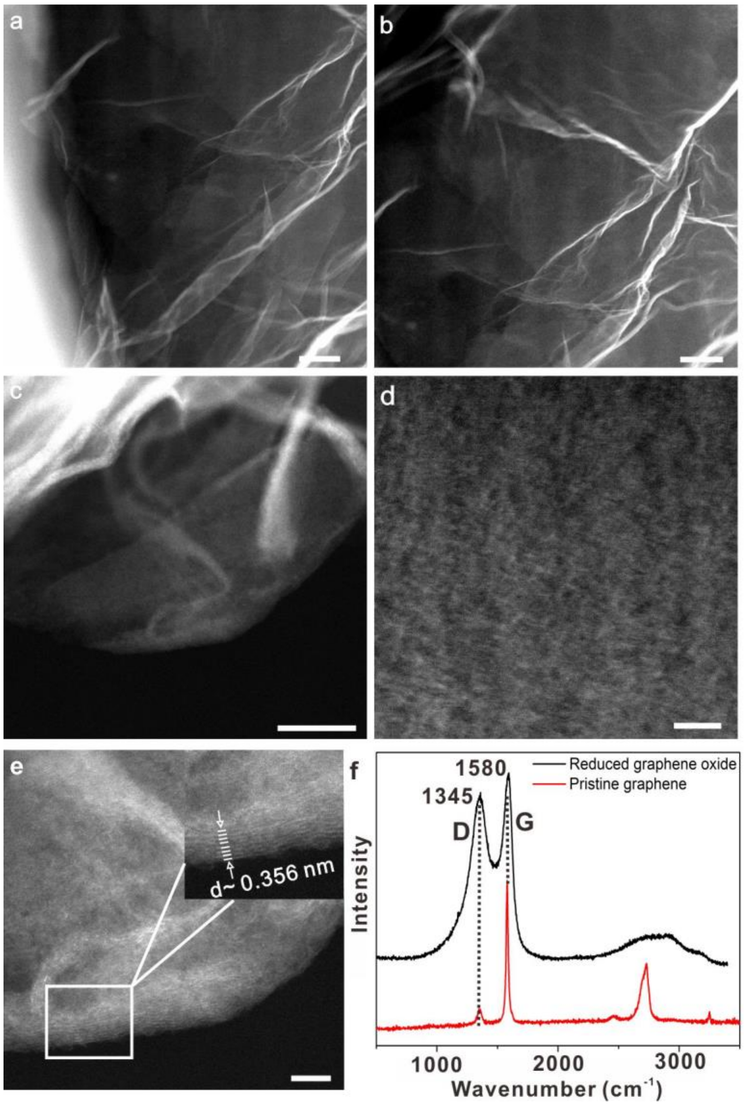

Supplementary Figure 1 | The physical properties of the reduced graphene oxide support. HAADF-STEM images of the reduced graphene oxide support at low (**a-c**) and high (**d-e**) magnifications. The inset in (**e**) shows the enlargement of the white rectangular area. Scale bars, 100 nm (**a**, **b**); 20 nm (**c**), 5 nm (**e**); 2 nm (**d**). (**f**) The Raman spectra of the pristine graphene and reduced graphene oxide support. Note: The high D band to G band ratio implies that there were small domains of the sp^2 carbons, along with a large amount of defects on the reduced graphene oxide[1].

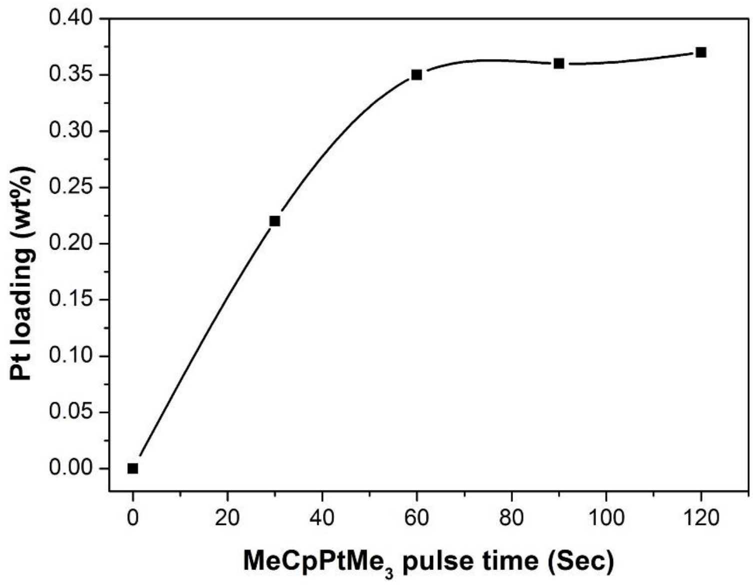

Supplementary Figure 2 | The Pt loadings in different 1cPt/graphene samples. These samples were synthesized with different $MeCpPtMe_3$ pulse time during Pt ALD, and the Pt loadings were determined by ICP-AES.

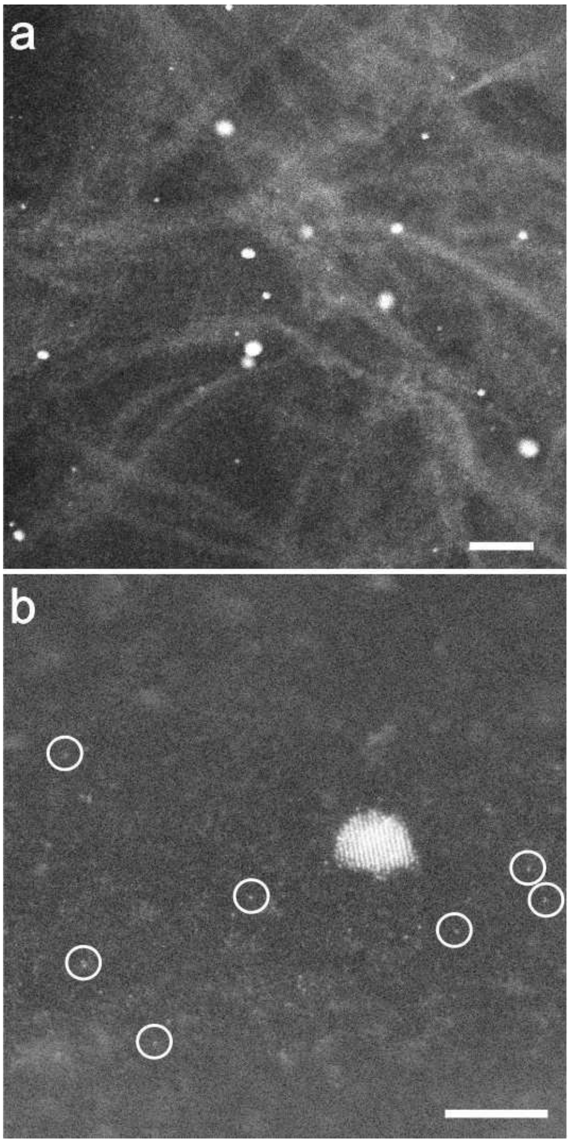

Supplementary Figure 3 │HAADF-STEM images of 2cPt/graphene. Some of Pt single atoms in (**b**) are highlighted by white cycles. Scale bars, 20 nm (**a**); 5 nm (**b**).

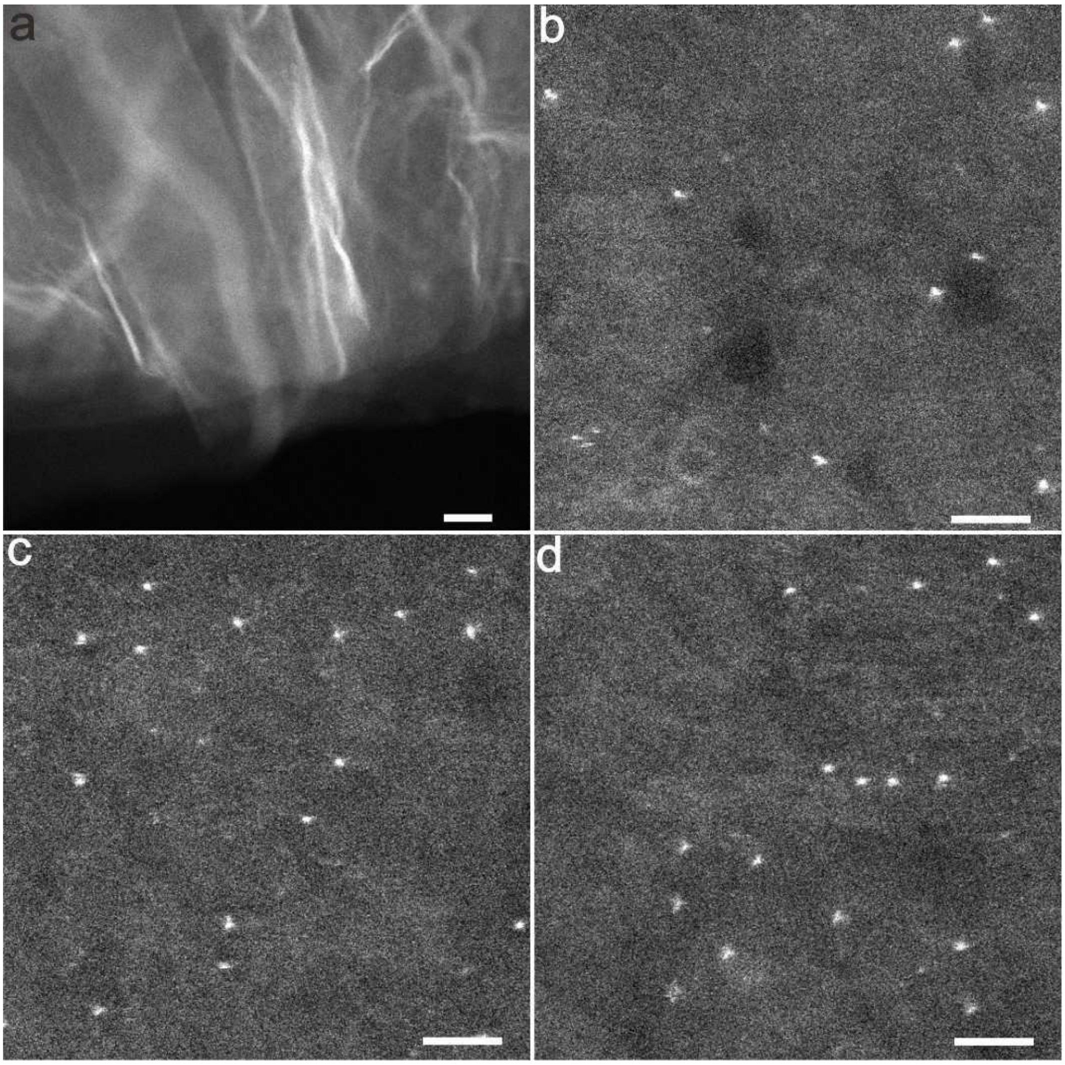

Supplementary Figure 4 │HAADF-STEM images of Pt_1/graphene at other locations. Scale bars, 20 nm (**a**), 2 nm (**b-d**).

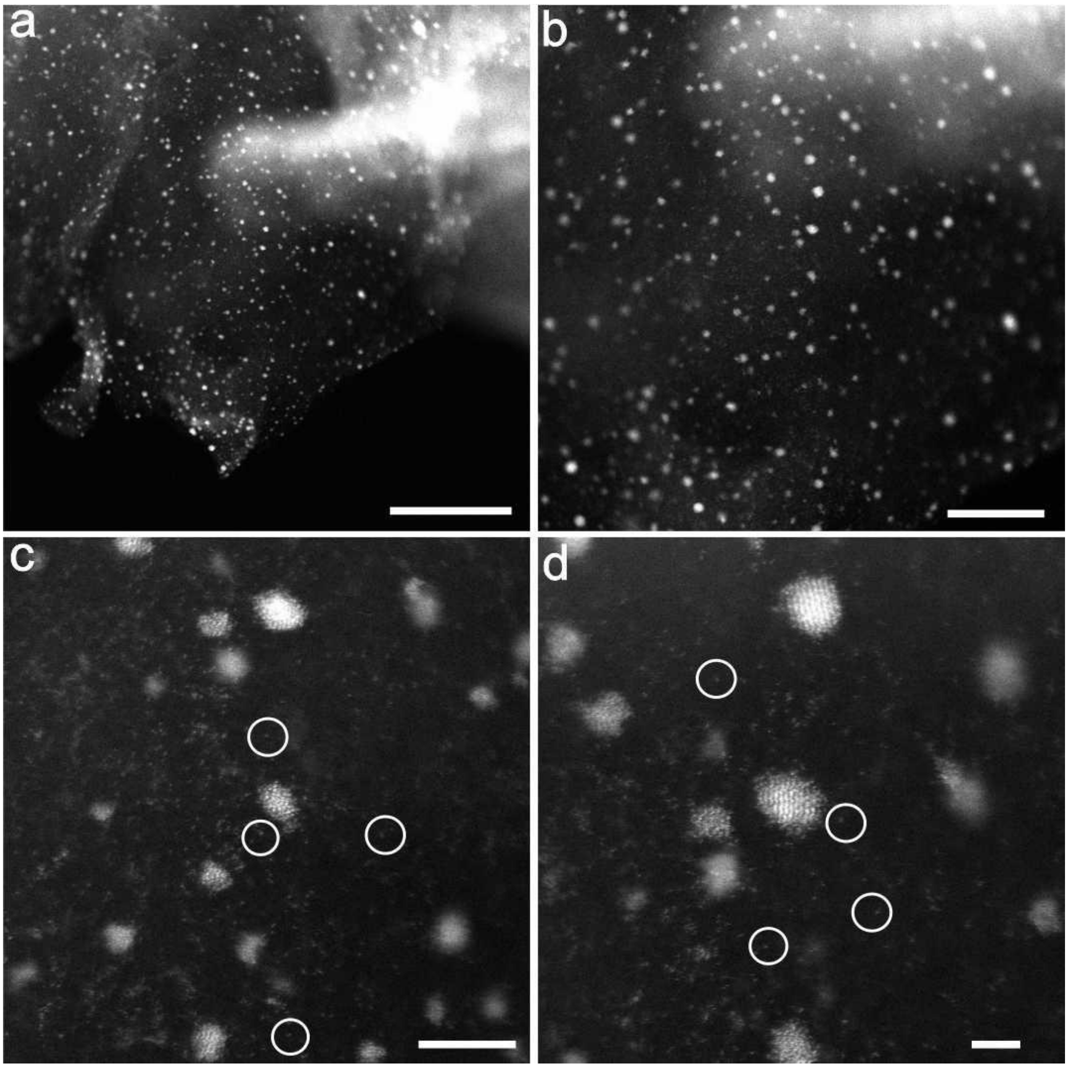

Supplementary Figure 5 | Morphology of an as-prepared 1cPt/graphene sample. Here the graphene support was obtained by thermal deoxygenation of graphene oxide at 700 ℃ for 30 sec under helium. Pt single atoms in (**c**) and (**d**) are highlighted by white cycles. Scale bars, 50 nm (**a**), 20 nm (**b**), 5 nm (**c**), 2 nm (**d**).

Supplementary Note 1:

When the graphene support was obtained by thermal deoxygenation of graphene oxide at 700 ℃ for 30 sec in helium, one cycle of Pt ALD at 250 ℃ would yield a mixture of both Pt nanoparticles and single atoms. Therefore, it is very critical to carefully tune the thermal reduction temperature to achieve proper nucleation sites for fabrication of highly uniform single-atom Pt_1/graphene catalyst.

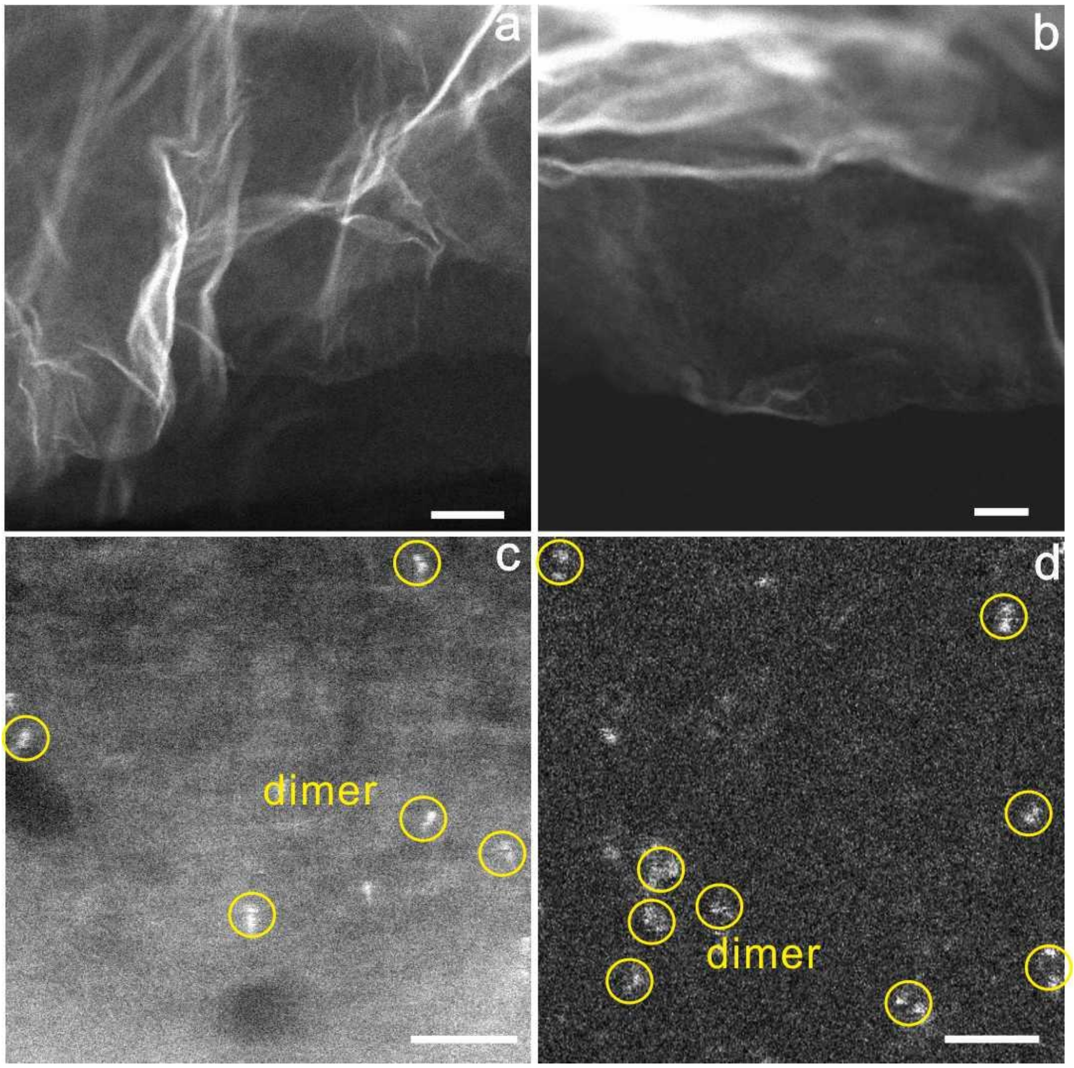

Supplementary Figure 6 | STEM images of Pt_2/graphene at other locations. Pt dimers in (**c**) and (**d**) are highlighted by yellow cycles. Scale bars, 50 nm (**a**), 20 nm (**b**), 2 nm (**c**), 1 nm (**d**).

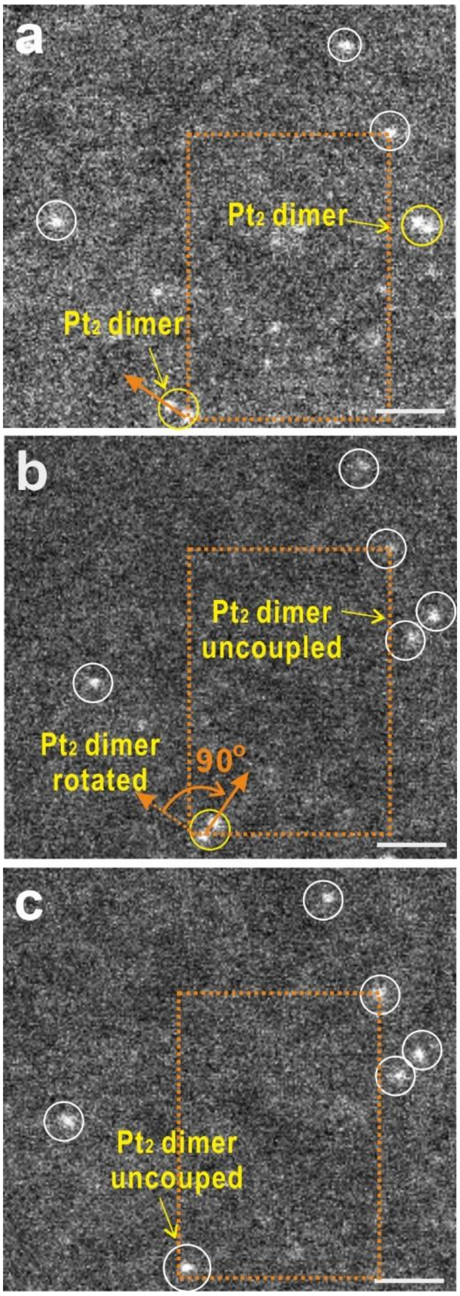

Supplementary Figure 7 | Morphology changes of Pt_2 dimers under electron beam. Rotating and uncoupling of Pt_2 dimers into two isolated Pt_1 single atoms by the electron beam were observed during STEM measurements. Scale bars, 1 nm (**a**-**c**). Pt_1 single atoms and Pt_2 dimers are highlighted by white and yellow circles, respectively.

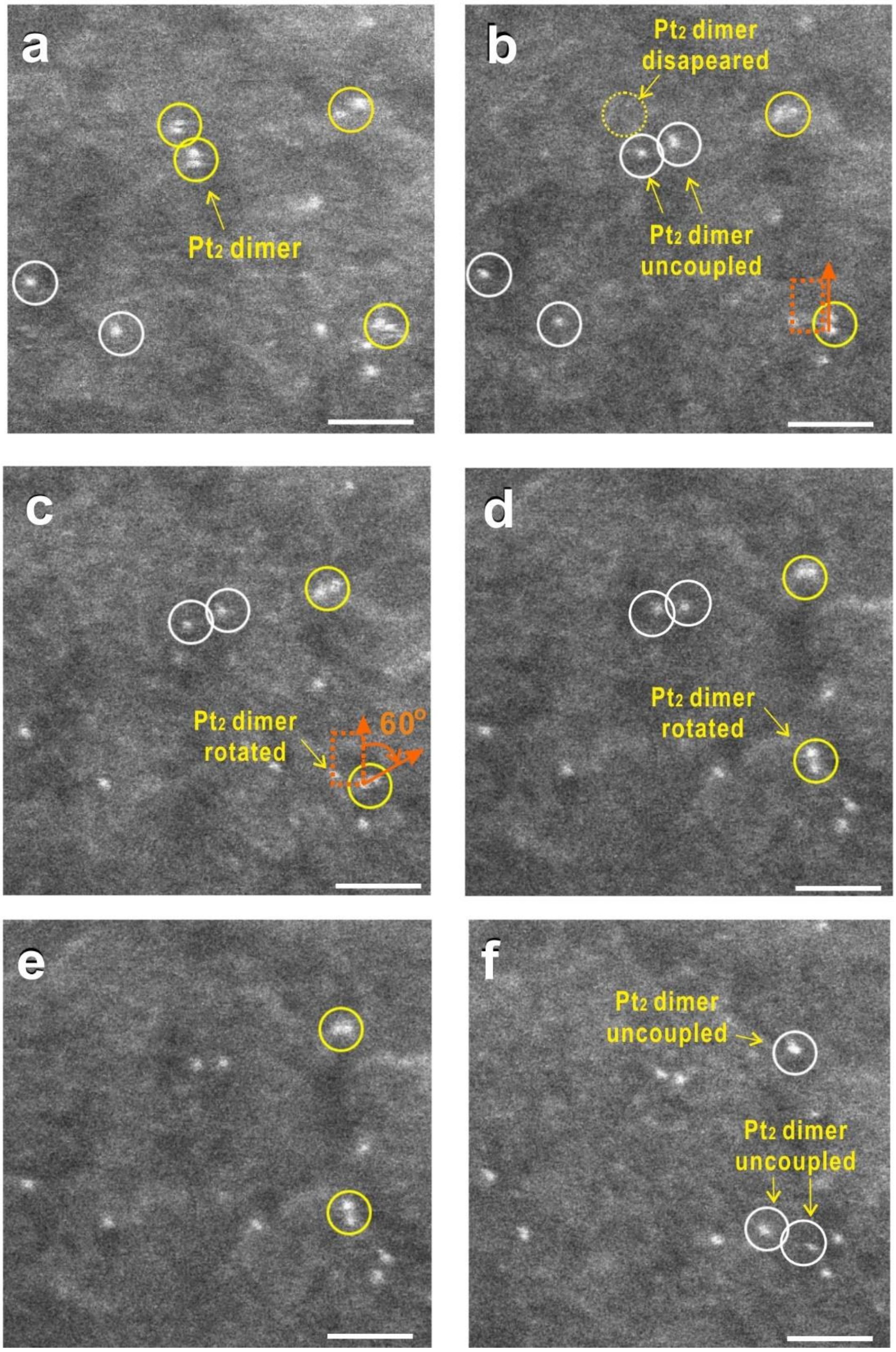

Supplementary Figure 8 | Morphology changes of Pt_2 dimers under electron beam. Rotating and uncoupling of Pt_2 dimers into two isolated Pt_1 single atoms by the electron beam were observed at different locations during STEM measurements. Scale bars, 2 nm (**a-f**). Pt_1 single atoms and Pt_2 dimers are highlighted by white and yellow circles, respectively.

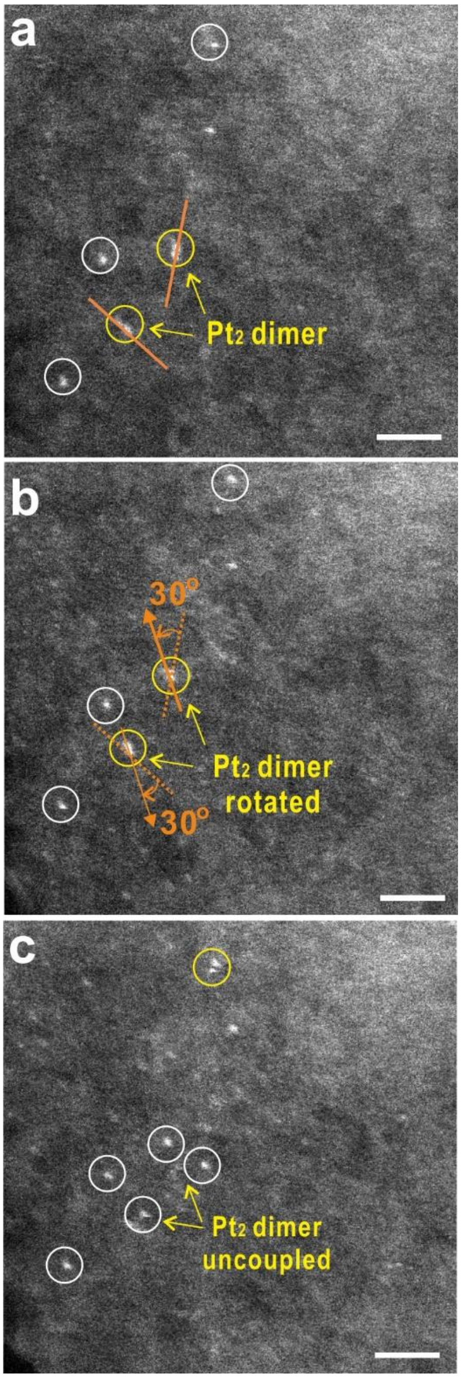

Supplementary Figure 9 | Morphology changes of Pt_2 dimers under electron beam. Rotating and uncoupling of Pt_2 dimers into two isolated Pt_1 single atoms by the electron beam were observed at different locations during STEM measurements. Scale bars, 2 nm (**a**-**c**). Pt_1 single atoms and Pt_2 dimers are highlighted by white and yellow circles, respectively.

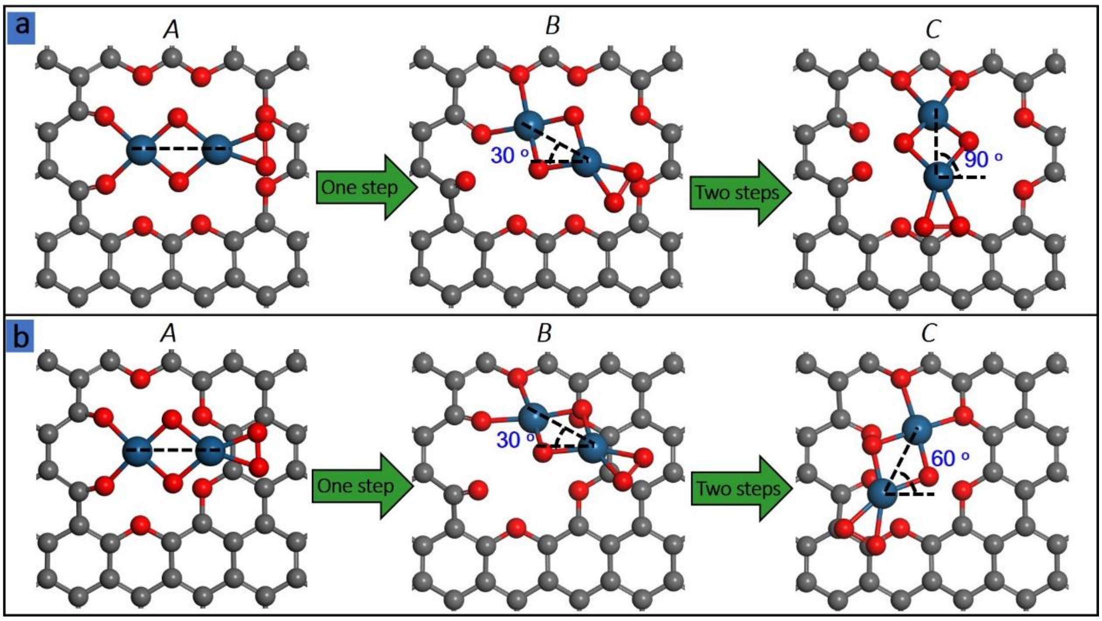

Supplementary Figure 10 │Speculated rotation of Pt_2O_x clusters along the graphene defect edge. (a) Walking along the edge of a carbon defect with a larger size, along with specific rotation angles of 30 ° and 90 °, for one and two steps walking, respectively. (b) Walking along the edge of a carbon defect a smaller size.

Supplementary Note 2:

Besides direct uncoupling of Pt_2 dimers, rotating and then uncoupling of Pt_2 dimers under electron beam were also frequently observed during STEM measurements (Supplementary Figures 7-9). In some cases, Pt_2 dimers were even directly evaporated from the surface (Supplementary Figure 8a, b). Such characteristic rotation by the specific angles under the electron beam might be related with the geometry of the graphene support and the size of carbon defect (Supplementary Figure 10).

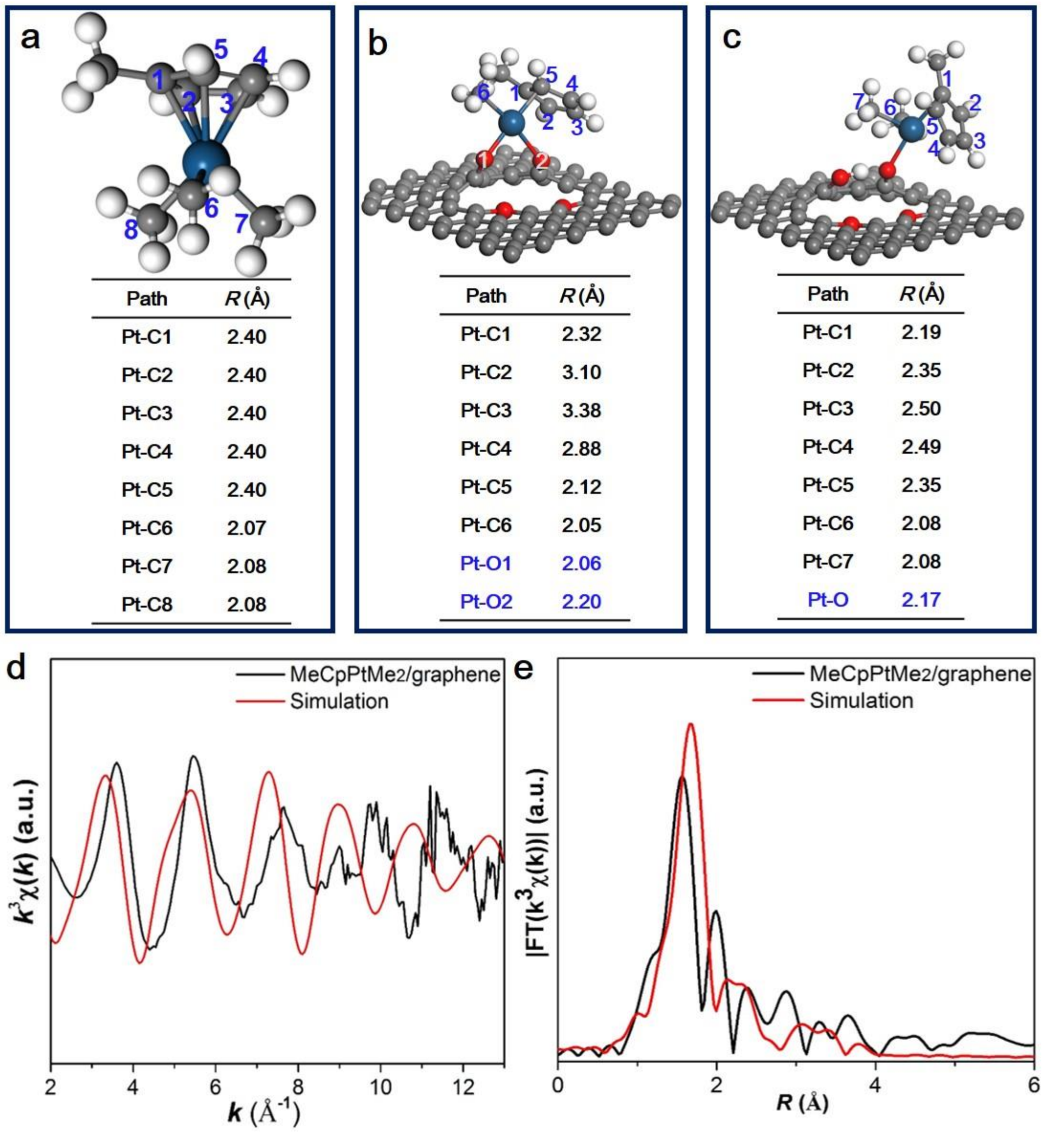

Supplementary Figure 11 │DFT calculated structures and XAFS spectra simulations. DFT calculated structures for MeCpPtMe$_3$ molecule (**a**), MeCpPtMe/graphene (**b**) and MeCpPtMe$_2$/graphene **(c).** The comparison of simulated EXAFS spectra based on the MeCpPtMe$_2$/graphene model in (**c**) with the experimental results in the *k*-space (**d**) and *R*-space (**e**), respectively. The ball in gray, white, red and blue represent carbon, hydrogen, oxygen and platinum, respectively. The information of the Pt-C and Pt-O bond lengths in the three structural models are also provided.

Supplementary Note 3:

The calculated bond lengths of Pt-C of the Pt-MeCp group in the MeCpPtMe$_3$ molecule agree well with the previous theoretical work, but slightly different from the experimental results.[2,3] When the MeCpPtMe$_3$ molecule reacts with one isolated phenol group, MeCpPtMe$_2$/graphene with one O at the interface is the expected structural model (Supplementary Figure 11c). However, simulated EXAFS spectrum based on this model does not fit the experimental data well in both *k*-space and *R*-space (Supplementary Figure 11c, d).

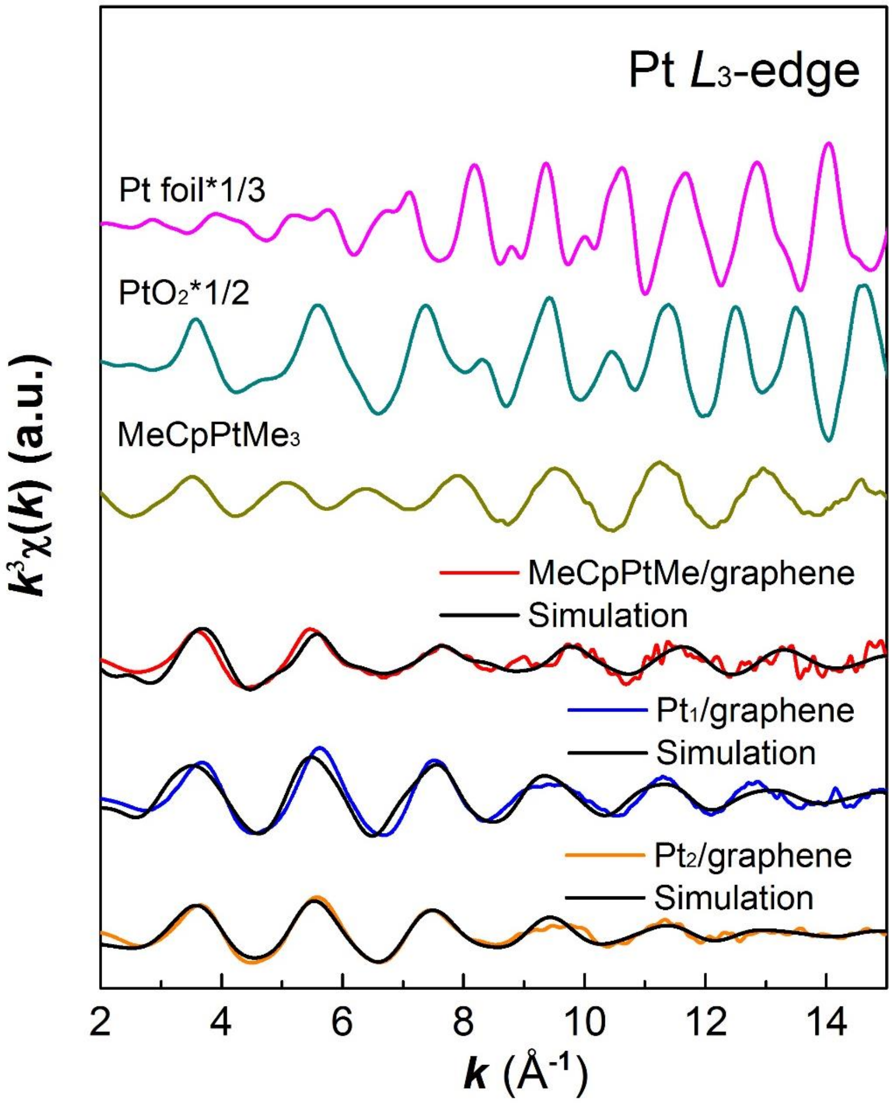

Supplementary Figure 12 │ k^3-weighted EXAFS oscillations and simulations. EXAFS oscillations and simulations of the MeCpPtMe/graphene, Pt_1/graphene and Pt_2/graphene samples at the Pt L_3-edge. The spectra of Pt foil, PtO_2 and $MeCpPtMe_3$ reference samples are also shown for comparison.

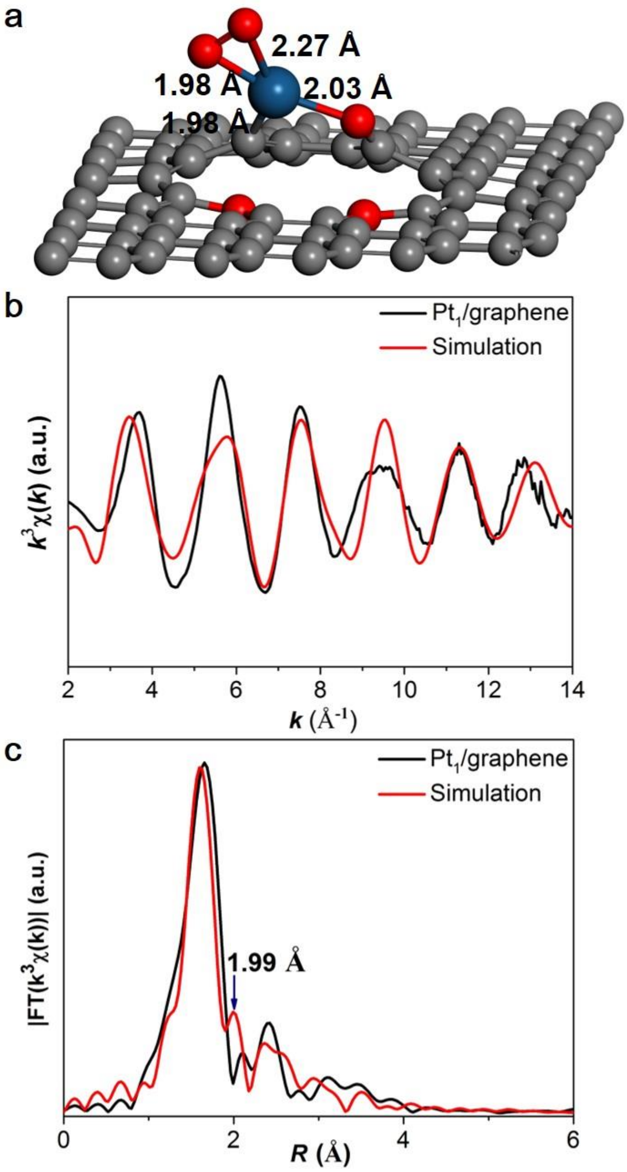

Supplementary Figure 13 │ A potential structure candidate for Pt_1/graphene. (**a**)The structure of this candidate with one C and one O at the interface. (**b, c**) Comparison of EXAFS simulations using this structure candidate with the experimental spectrum. A split peak at 1.99 Å was observed owing to the elongated Pt-O bond of 2.27 Å. The balls in gray, red and blue represent carbon, oxygen and platinum, respectively.

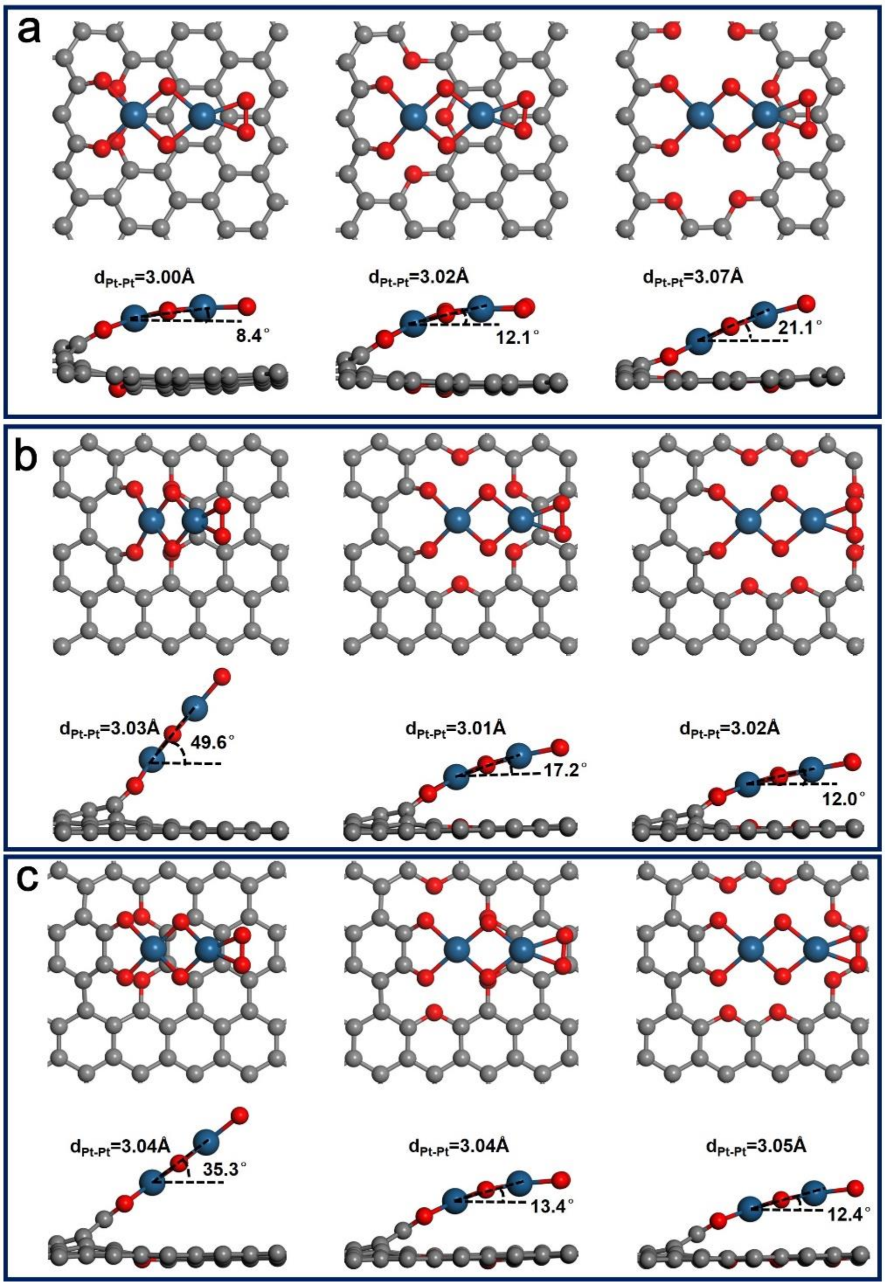

Supplementary Figure 14 │ DFT suggested structures for Pt_2/graphene. Top and side views of the optimized structures for Pt_2 dimers on graphene with different size of carbon vacancies and interfacial structures. (**a**) Two neighbor sites along the zigzag edge; (**b**) two second nearest sites along the armchair edge, (**c**) two neighbor sites along the armchair edge. The size of vacancies increase from left to right in each subgraph. The tilted angles and the distance between two Pt atoms are labeled. The balls in gray, red and blue represent carbon, oxygen and platinum, respectively.

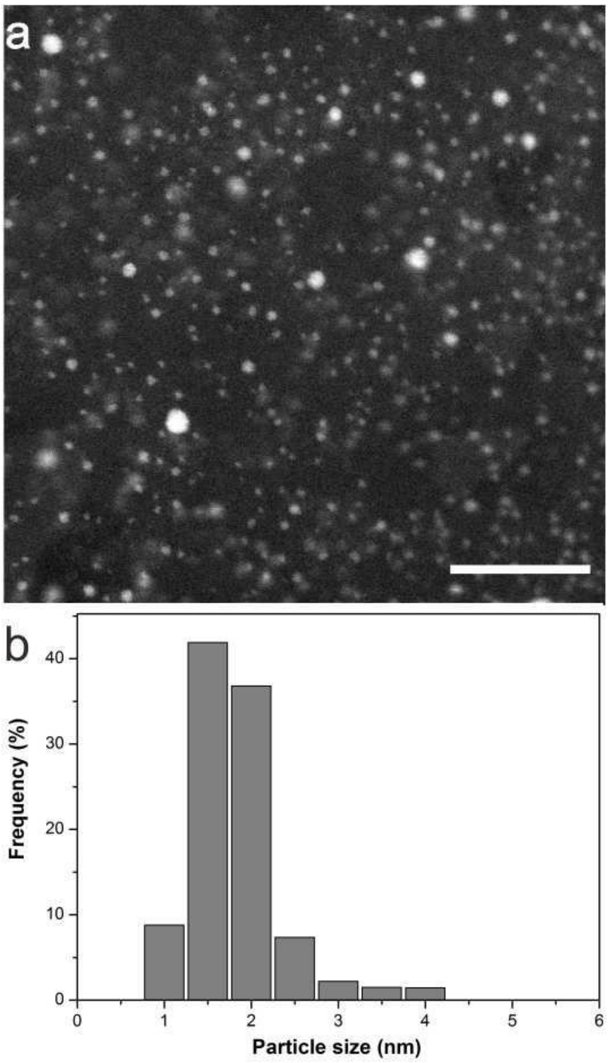

Supplementary Figure 15 │ Morphology of the Pt/graphene-WI catalyst. (**a**) Representative aberration-corrected HAADF-STEM image of the Pt/graphene-WI sample. Scale bar, 20 nm. (**b**) The particle size distribution histogram of Pt nanoparticles in this sample. The Pt particle size is about 1.8 ± 0.5 nm in average.

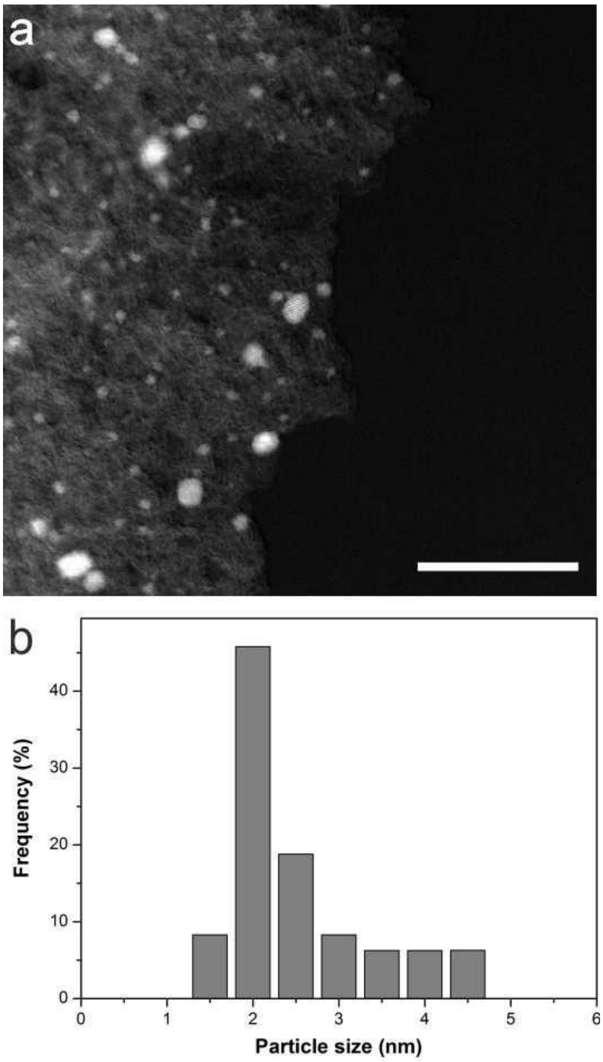

Supplementary Figure 16 │ Morphology of the commercial Pt/carbon catalyst. (**a**) Representative aberration-corrected HAADF-STEM image of the Pt/carbon sample. Scale bar, 20 nm. (**b**) The particle size distribution histogram of Pt nanoparticles in this sample. The Pt particle size is about 2.3 ± 0.7 nm in average.

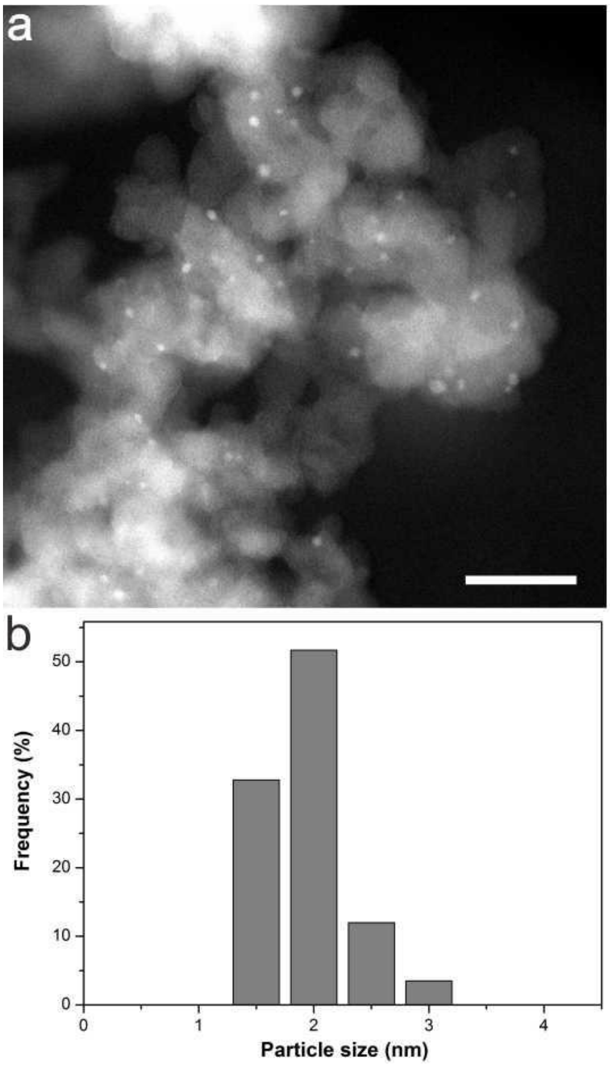

Supplementary Figure 17 │ Morphology of the Pt/SiO_2 ALD catalyst. (**a**) Representative aberration-corrected HAADF-STEM image of the Pt/SiO_2 sample. Scale bar, 20 nm. (**b**) The particle size distribution histogram of Pt nanoparticles in this sample. The particle size is about 1.9 ±0.3 nm in average.

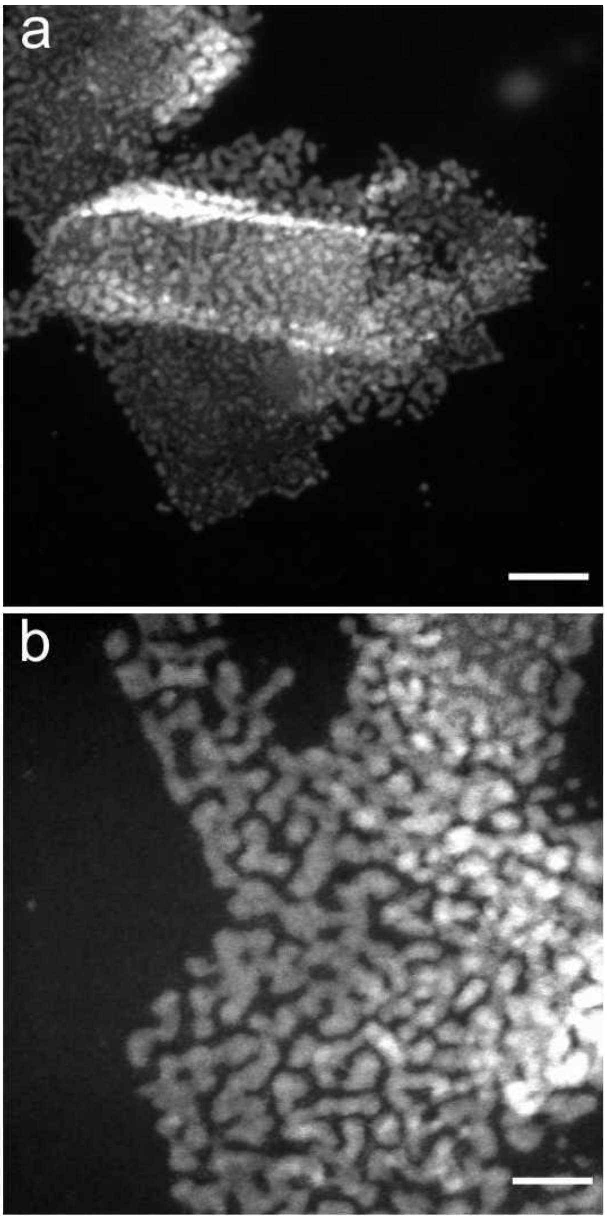

Supplementary Figure 18 │ Morphology of the commercial PtO_2 catalyst. Scale bars, 20 nm (**a**); 10 nm (**b**).

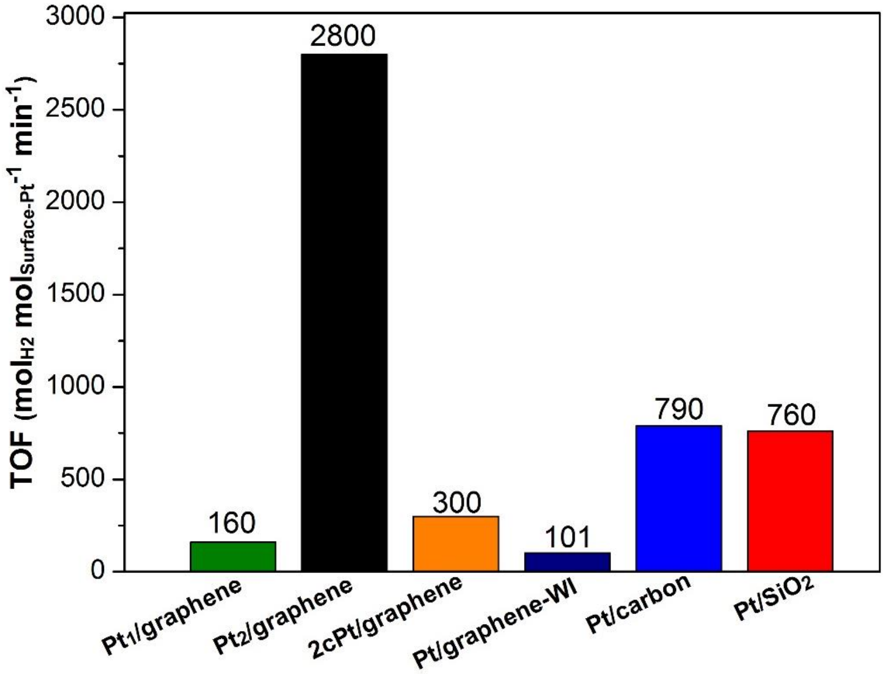

Supplementary Figure 19 │ TOFs of various Pt catalysts in the AB hydrolysis reaction. The TOFs are calculated according to the mole of surface Pt sites. Note: the Pt dispersions of the Pt NP catalysts were calculated using the equation of $d_{Pt} = (1.1 / D_{Pt}) \times 100$, d_{Pt} is the Pt dispersion, D_{Pt} is the Pt particle size.

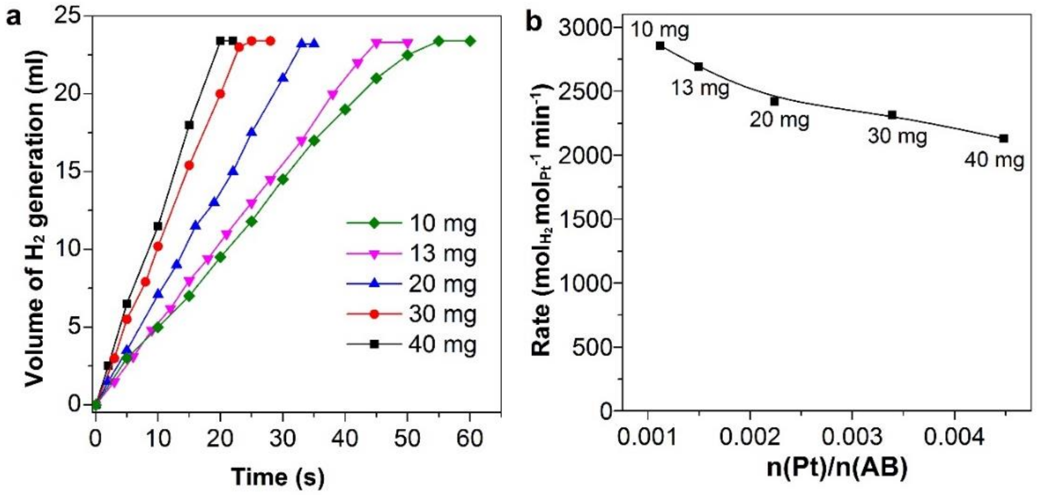

Supplementary Figure 20 │ Catalytic performance of Pt_2/graphene in the AB hydrolysis reaction. (a) Plots of time versus volume of hydrogen generation by the dimeric Pt_2/graphene catalyst with different catalyst amounts. (**b)** The mass specific rates of Pt_2/graphene at the different mole ratios of n(Pt)/n(AB).

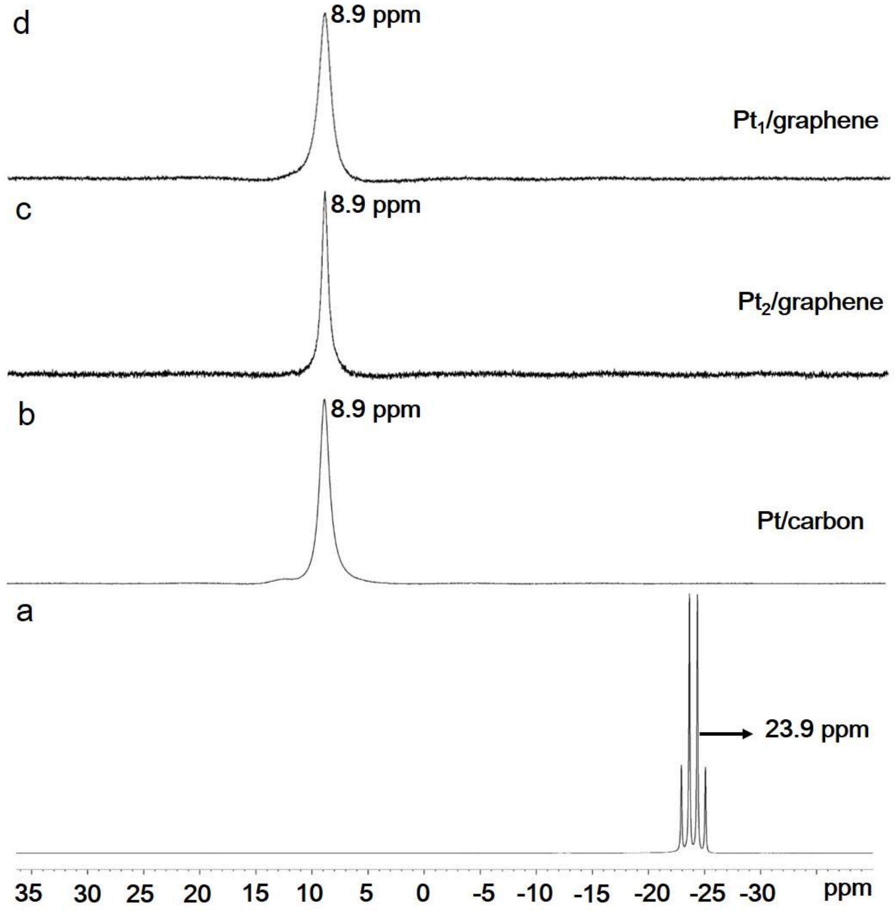

Supplementary Figure 21 │ ^{11}B NMR spectra of the AB aqueous solution before and after reaction. (a) The fresh AB aqueous solution; the solution after reaction over Pt/carbon (b), Pt_2/graphene (c) and Pt_1/graphene (d), respectively. Note: D_2O was used in these cases.

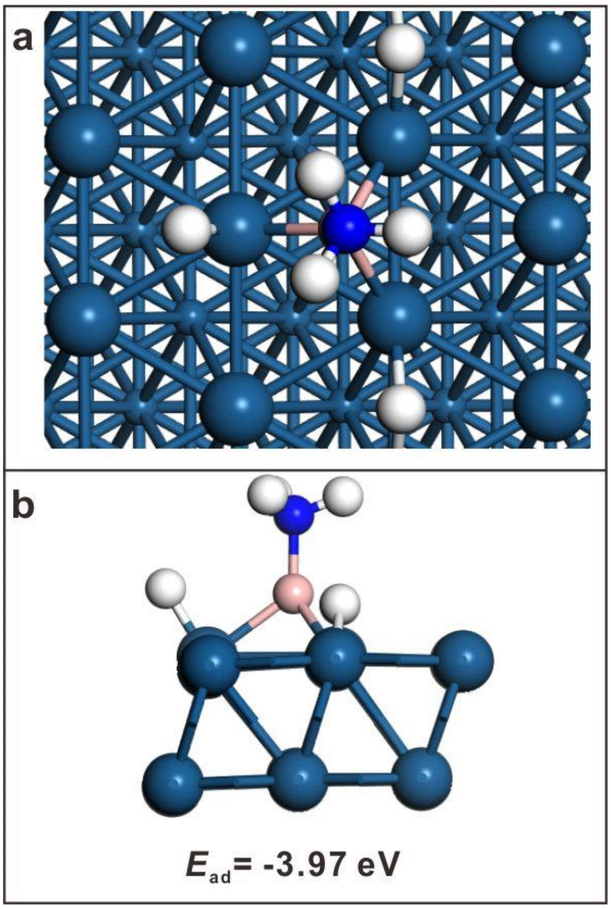

Supplementary Figure 22 │ Optimized adsorption of AB on Pt (111) surface. (a) top view; **(b)** side views. The ball in white, pink, blue and dark blue represent hydrogen, boron, nitrogen and platinum, respectively.

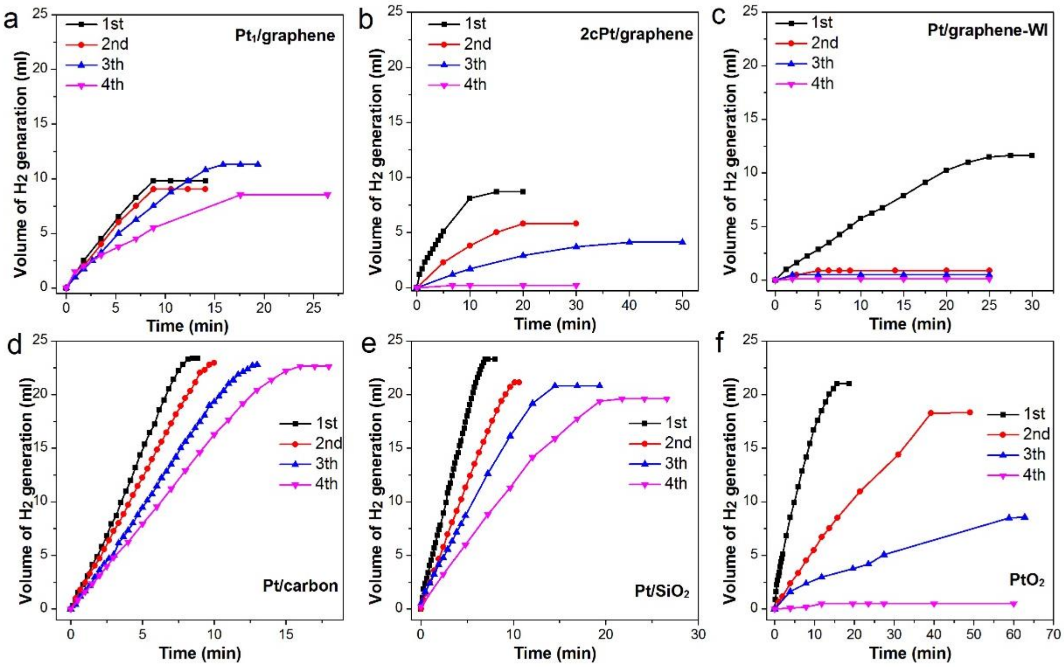

Supplementary Figure 23 │ Recycling tests over various Pt catalysts in the AB hydrolysis reaction. (**a**) Pt_1/graphene; (**b**) 2cPt/graphene; (**c**) Pt/graphene-WI; (**d**) Pt/carbon; (**e**) Pt/SiO_2; and (**f**) PtO_2. Note: After each run, additional 0.325 mmol of pure AB was added into the reaction flask.

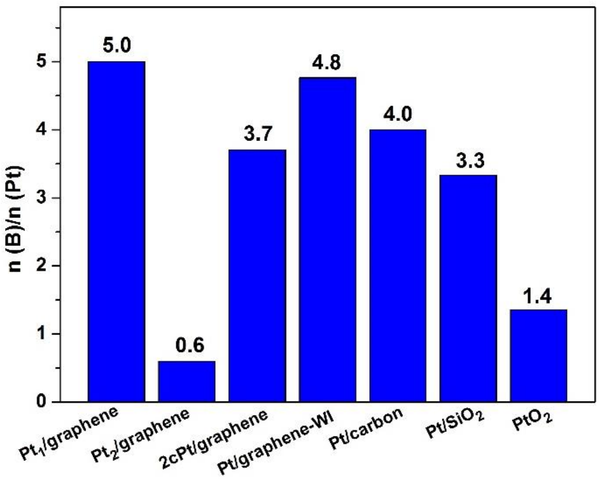

Supplementary Figure 24 │ The mole ratio of B element to Pt surface atoms in all used samples after four recycling test determined by ICP-AES.

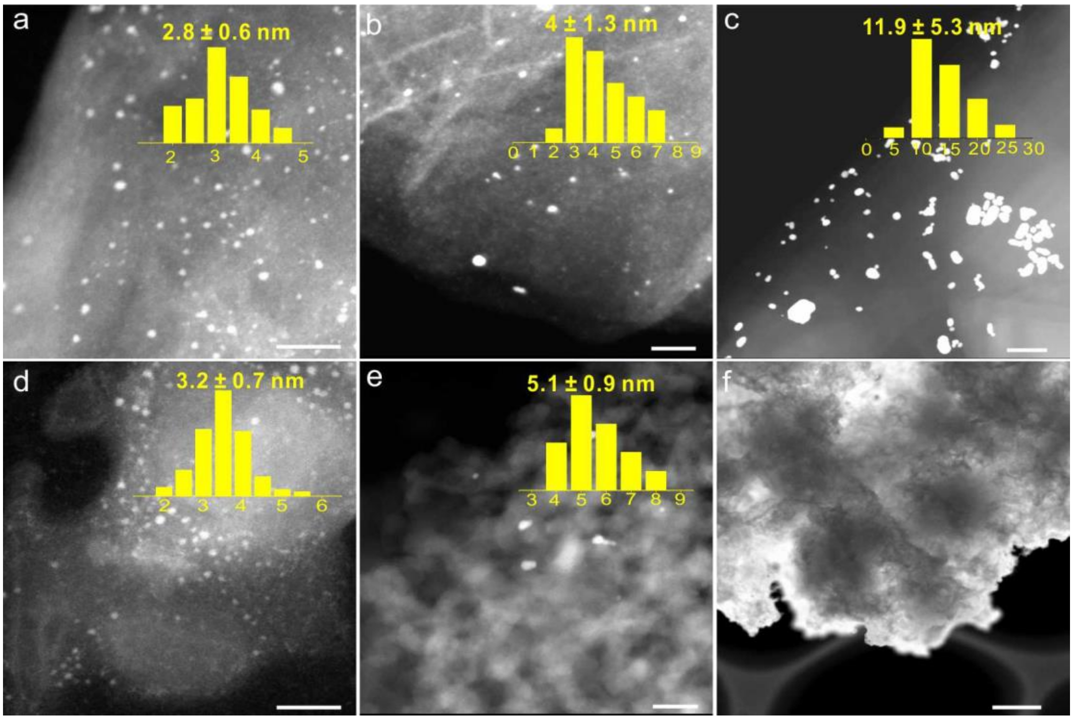

Supplementary Figure 25 │ Morphologies of used Pt catalysts after the recycling test. (**a**) Pt_1/graphene, scale bar, 20 nm; (**b**) 2cPt/graphene, scale bar, 20 nm; (**c**) Pt/graphene-WI, scale bar, 50 nm; (**d**) Pt/carbon, scale bar, 20 nm; (**e**) Pt/SiO_2, scale bar, 20 nm; (**f**) PtO_2. Scale bar, 500 nm.

Supplementary Tables

Supplementary Table 1| The Pt loadings in different 1cPt/graphene samples determined by ICP-AES. Here these 1cPt/graphene samples were prepared by depositing one cycle of Pt ALD on several different graphene supports at either 250 or 300 ℃. These supports were obtained by thermal deoxygenation of graphene oxide at different temperatures with different time in helium at a flow rate of 50 mL/min.

Annealing temperature (℃)	Annealing time (min)	ALD temperature (℃)	Pt loadings (wt%)
-[a]	-	250	0.01
-[b]	-	250	0.02
700	0.5	250	2.2
1050	1	250	0.9
1050	2	250	0.3
1050	2	250	0.31
1050	5	250	0.02
1050	5	300	2.4
1050	10	250	0.01

[a] Pristine graphene without any treatment.

[b] Pristine graphene was pretreated by O_2 at 250 ℃ for 10 min.

Supplementary Note 4:

Clearly, Pt does not nucleate on either pristine graphene or the pristine graphene pretreated by O_2 at 250 ℃. In other words, exposing graphene to O_2 would not be able to create any additional nucleation sites for Pt ALD at 250 ℃.

On the other hand, thermal deoxygenation of graphene oxide at 1050 ℃ for more than 5 min will remove all the oxygen functional groups from the graphene surface, thus the nucleation of Pt is inhibited, which is very similar to our recent work of Pd ALD on reduced graphene[4]. However, when Pt ALD was performed on the same graphene support at 300 ℃ for one cycle, we found that the Pt loading was as high as 2.4 wt% (**Supplementary Table 1**). Clearly, the nucleation of Pt at the graphene defect sites was feasible at 300 ℃, in a good agreement with the literature where selective deposition of Pt by ALD at graphene line defects was reported[5].

Supplementary Table 2 | The Pt loadings in various ALD samples grown on different supports with one or two cycles of Pt ALD.

Support	T (℃)	Trials	Pt loadings (wt%) and morphology from different ALD cycles				Ratio of Pt loadings[a]	Notes
			One cycle	Morphology	Two cycles	Morphology		
Reduced graphene	250	1	**0.27**	Single atoms	**0.51**	Dimers [b]	**1.9**	This work
		2	**0.2**	Single atoms	**0.4**	Dimers [b]	**2**	This work
		3	**0.19**	Single atoms	**0.36**	Dimers [b]	**1.9**	This work
		4	**0.24**	Single atoms	**0.5**	Dimers [b]	**2.08**	This work
		5	**0.3**	Single atoms	**0.6**	Dimers [b]	**2**	This work
		6	**0.35**	Single atoms	**0.71**	Dimers [b]	**2.02**	This work
		7	**0.2**	Single atoms	**0.41**	Dimers [b]	**2.05**	This work
		8	**0.41**	Single atoms	**0.78**	Dimers [b]	**1.9**	This work
		9	**0.3**	Single atoms	**0.6**	Dimers [b]	**2**	This work
Al_2O_3	290	-	**0.7**	NPs	**2.9**	NPs	**4.1**	This work
Reducd graphene	250	-	**0.38**	Single atoms	**0.71**	NPs	**1.87**	This work
Al_2O_3	300	-	**2.5**	NPs	**6.5**	NPs	**2.3**	Ref [6]
$SrTiO_3$	300	-	**10**	NPs	**23**	NPs	**2.3**	Ref [7]

[a]The Pt loading from two ALD cycles to the one from one ALD cycle.
[b]The second Pt ALD cycle was performed at 150 ℃.

Supplementary Note 5:

Regarding bottom-up synthesis of Pt_2 dimers, each Pt_1 single atom anchors only one $MeCpPtMe_3$ precursor in the second Pt ALD cycle. Therefore, the loading of the dimeric Pt_2 sample is twice higher than that of the single-atom Pt_1 sample. When the nucleation sites are not Pt_1 single atoms, e.g. Pt nanoparticles, there would be no any obvious relation between the Pt loadings from the samples by one ALD cycle and the samples by two ALD cycles.

Supplementary Table 3 | The Pt loadings in various Pt catalysts used in the hydrolytic dehydrogenation of AB reaction test.

Samples	Pt loadings (wt %)
Pt_1/graphene	0.35
Pt_2/graphene	0.72
2cPt/graphene	0.71
Pt/graphene-WI	3.0
Pt/carbon	5.0
Pt/SiO_2	0.5

Supplementary Table 4 | A comparison of catalytic performances of various supported metal catalysts in hydrolytic dehydrogenation of AB.

Samples	Metal particle size (nm)	n(Pt)/n(AB) ratio	Reaction Temp. (℃)	Mass specific rate ($mol_{H2}\ mol_{metal}^{-1}\ min^{-1}$)	Notes
**Pt_2/graphene**	**-**	**1.1×10^{-3}**	**27**	**2800**	**This work**
**Pt_2/graphene**	**-**	**2.3×10^{-3}**	**27**	**2421**	**This work**
**Pt_2/graphene**	**-**	**4.5×10^{-3}**	**27**	**2129**	**This work**
Pt_1/graphene	-	1.1×10^{-3}	27	160	This work
2cPt/graphene	-[a]	1.1×10^{-3}	27	110	This work
Pt/graphene(WI)	1.8	1.1×10^{-3}	27	62	This work
Pt/carbon	2.3	1.1×10^{-3}	27	380	This work
Pt/SiO_2	1.9	1.1×10^{-3}	27	440	This work
Pt/CNTs-O-HT	1.3	4.7×10^{-3}	30	567	Ref [8]
Pt/AC	3	1.8×10^{-3}	30	290	Ref [8]
Pt/CNTS	1.9	2.4×10^{-3}	25	416	Ref [9]
Pt/γ-Al_2O_3	1.5	1.8×10^{-2}	25	222	Ref [10]
Pt/SiO_2	5.1	1.8×10^{-2}	25	56	Ref [10]
Pt/MIL-101	1.8	1.4×10^{-3}	25	1020	Ref [11]
Pt/CNTs	1.8	4.7×10^{-3}	25	410	Ref [12]
Pt/C	1.9	1.8×10^{-2}	25	111	Ref [13]
Pt/CeO_2	3	1.8×10^{-2}	25	182	Ref [14]
Ru/carbon	7.0	7.4×10^{-4}	25	670	Ref [15,16]
Ru/TiO_2	1.7	1.0×10^{-3}	25	604	Ref [16]
Ru/graphene	1.9	2.0×10^{-3}	25	600	Ref [17]
Ru/HfO_2	3.5	4.0×10^{-3}	25	170	Ref [18]
Rh/γ-Al_2O_3	2.5	1.8×10^{-2}	25	128	Ref [10]
Pd/MIL-101	1.8	1.9×10^{-2}	25	45	Ref [19]
Pd/CeO_2	2~6	1.1×10^{-2}	25	29	Ref [20]
Pd/graphene	5.0	5.6×10^{-2}	25	9.7	Ref [21]
Ni/CNT	5.6	2.5×10^{-2}	25	26.2	Ref [22]
Ni/MSC-30	6.3	1.6×10^{-2}	25	30.7	Ref [23]
RuNi/TiO_2	2.3	1.0×10^{-3}	25	703	Ref [16]
RuNi/MIL-101	1.8	1.7×10^{-2}	25	272	Ref [24]
RuNi/$Ti_3C_2X_2$	3.7	1.5×10^{-2}	25	82.4	Ref [25]
PdNi/CS	2.5	2.4×10^{-2}	25	182	Ref [26]
$Cu_{0.5}Ni_{0.5}$/ MCNS	6.7	3.6×10^{-2}	25	23.5	Ref [27]
$Cu_{0.8}Co_{0.2}O$/GO	3	2.4×10^{-2}	25	70	Ref [28]
CuCo/C_3N_4	-	2.0×10^{-2}	25	75	Ref [29]
NiMo/graphene	10.4	5.0×10^{-2}	25	67	Ref [30]
AuNi/MOF	1.8	1.7×10^{-2}	25	66	Ref [31]
PtRu/C	2	3×10^{-2}	25	8	Ref [32]

[a]A mixture of Pt single atoms and nanoparticles.

Supplementary References:

1 Yang, D. *et al.* Chemical analysis of graphene oxide films after heat and chemical treatments by X-ray photoelectron and Micro-Raman spectroscopy. *Carbon* **47**, 145-152 (2009).

2 Shen, J., Muthukumar, K., Jeschke, H. O. & Valentí, R. Physisorption of an organometallic platinum complex on silica: anab initiostudy. *New J. Phys.* **14**, 073040 (2012).

3 Xue, Z. *et al.* Characterization of (methylcyclopentadienyl) trimethylplatinum and low-temperature organometallic chemical vapor deposition of platinum metal. *J. Am. Chem. Soc.* **111**, 8779-8784 (1989).

4 Yan, H. *et al.* Single-Atom Pd-1/Graphene Catalyst Achieved by Atomic Layer Deposition: Remarkable Performance in Selective Hydrogenation of 1,3-Butadiene. *J. Am. Chem. Soc.* **137**, 10484-10487 (2015).

5 Kim, K. *et al.* Selective metal deposition at graphene line defects by atomic layer deposition. *Nat. Commun.* **5**, 4781 (2014).

6 Setthapun, W. *et al.* Genesis and Evolution of Surface Species during Pt Atomic Layer Deposition on Oxide Supports Characterized by in Situ XAFS Analysis and Water-Gas Shift Reaction. *J. Phys. Chem. C* **114**, 9758-9771 (2010).

7 Christensen, S. T. *et al.* Controlled Growth of Platinum Nanoparticles on Strontium Titanate Nanocubes by Atomic Layer Deposition. *Small* **5**, 750-757 (2009).

8 Chen, W. Y. *et al.* Unique reactivity in Pt/CNT catalyzed hydrolytic dehydrogenation of ammonia borane. *Chem. Commun.* **50**, 2142-2144 (2014).

9 Zhang, J. *et al.* Highly dispersed Pt nanoparticles supported on carbon nanotubes produced by atomic layer deposition for hydrogen generation from hydrolysis of ammonia borane. *Catal. Sci. Technol.* **7**, 322-329 (2017).

10 Chandra, M. & Xu, Q. Room temperature hydrogen generation from aqueous ammonia-borane using noble metal nano-clusters as highly active catalysts. *J. Power Sources* **168**, 135-142 (2007).

11 Aijaz, A. *et al.* Immobilizing highly catalytically active Pt nanoparticles inside the pores of metal-organic framework: a double solvents approach. *J. Am. Chem. Soc.* **134**, 13926-13929 (2012).

12 Chen, W. Y. *et al.* Mechanistic Insight into Size-Dependent Activity and Durability in Pt/CNT Catalyzed Hydrolytic Dehydrogenation of Ammonia Borane. *J. Am. Chem. Soc.* **136**, 16736-16739 (2014).

13 Xu, Q. & Chandra, M. A portable hydrogen generation system: Catalytic hydrolysis of ammonia–borane. *J. Alloys Compd.* **446-447**, 729-732 (2007).

14 Wang, X., Liu, D., Song, S. & Zhang, H. Synthesis of highly active Pt-CeO2 hybrids with tunable secondary nanostructures for the catalytic hydrolysis of ammonia borane. *Chem. Commun.* **48**, 10207-10209 (2012).

15 Navlani-García, M., Mori, K., Nozaki, A., Kuwahara, Y. & Yamashita, H. Highly efficient Ru/carbon catalysts prepared by pyrolysis of supported Ru complex towards the hydrogen production from ammonia borane. *Appl. Catal. A Gen.* **527**, 45-52 (2016).

16 Mori, K., Miyawaki, K. & Yamashita, H. Ru and Ru–Ni Nanoparticles on TiO2Support as Extremely Active Catalysts for Hydrogen Production from Ammonia–Borane. *Acs. Catal.* **6**, 3128-3135 (2016).

17 Du, C. *et al.* Facile synthesis of monodisperse ruthenium nanoparticles supported on graphene for hydrogen generation from hydrolysis of ammonia borane. *Int. J. Hydrogen Energy* **40**, 6180-6187 (2015).

18 Kalkan, E. B., Akbayrak, S. & Özkar, S. Ruthenium(0) nanoparticles supported on nanohafnia: A highly active and long-lived catalyst in hydrolytic dehydrogenation of ammonia borane. *J. Mol. Catal. A* **430**, 29-35 (2016).

19 Dai, H., Su, J., Hu, K., Luo, W. & Cheng, G. Pd nanoparticles supported on MIL-101 as high-performance catalysts for catalytic hydrolysis of ammonia borane. *Int. J. Hydrogen Energy* **39**, 4947-4953 (2014).

20 Tonbul, Y., Akbayrak, S. & Özkar, S. Palladium(0) nanoparticles supported on ceria: Highly active and reusable catalyst in hydrogen generation from the hydrolysis of ammonia borane. *Int. J. Hydrogen Energy* **41**, 11154-11162 (2016).

21 Zhong, W.-d. *et al.* Active 3D Pd/graphene aerogel catalyst for hydrogen generation from the hydrolysis of ammonia-borane. *Int. J. Hydrogen Energy* **41**, 15225-15235 (2016).

22 Zhang, J. *et al.* Ni nanoparticles supported on CNTs with excellent activity produced by atomic layer deposition for hydrogen generation from the hydrolysis of ammonia borane. *Catal. Sci. Technol.* **6**, 2112-2119 (2016).

23 Li, P. Z., Aijaz, A. & Xu, Q. Highly dispersed surfactant-free nickel nanoparticles and their remarkable catalytic activity in the hydrolysis of ammonia borane for hydrogen generation. *Angew. Chem. Int. Ed.* **51**, 6753-6756 (2012).

24 Roy, S., Pachfule, P. & Xu, Q. High Catalytic Performance of MIL-101-Immobilized NiRu Alloy Nanoparticles towards the Hydrolytic Dehydrogenation of Ammonia Borane. *Eur. J. Inorg. Chem.* **2016**, 4353-4357 (2016).

25 Li, X., Zeng, C. & Fan, G. Ultrafast hydrogen generation from the hydrolysis of ammonia borane catalyzed by highly efficient bimetallic RuNi nanoparticles stabilized on Ti3C2X2 (X = OH and/or F). *Int. J. Hydrogen Energy* **40**, 3883-3891 (2015).

26 Shang, N. *et al.* Synergetic catalysis of NiPd nanoparticles supported on biomass-derived carbon spheres for hydrogen production from ammonia borane at room temperature. *Int. J. Hydrogen Energy* **42**, 5733-5740 (2016).

27 Yen, H., Seo, Y., Kaliaguine, S. & Kleitz, F. Role of Metal–Support Interactions, Particle Size, and Metal–Metal Synergy in CuNi Nanocatalysts for H2Generation. *Acs. Catal.* **5**, 5505-5511 (2015).

28 Feng, K. *et al.* CuxCo1−xO Nanoparticles on Graphene Oxide as A Synergistic Catalyst for High-Efficiency Hydrolysis of Ammonia–Borane. *Angew. Chem. Int. Ed.* **55**, 11950-11954 (2016).

29 Zhang, H. *et al.* Highly efficient visible-light-driven catalytic hydrogen evolution from ammonia borane using non-precious metal nanoparticles supported by graphitic carbon nitride. *J. Mater. Chem. A* **5**, 2288-2296 (2016).

30 Yao, Q., Lu, Z.-H., Huang, W., Chen, X. & Zhu, J. High Pt-like activity of the Ni–Mo/graphene catalyst for hydrogen evolution from hydrolysis of ammonia borane. *J. Mater. Chem. A* **4**, 8579-8583 (2016).

31 Zhu, Q. L., Li, J. & Xu, Q. Immobilizing metal nanoparticles to metal-organic frameworks with size and location control for optimizing catalytic performance. *J. Am. Chem. Soc.* **135**, 10210-10213 (2013).

32 Yao, C., Zhuang, L., Cao, Y., Ai, X. & Yang, H. Hydrogen release from hydrolysis of borazane on Pt- and Ni-based alloy catalysts. *Int. J. Hydrogen Energy* **33**, 2462-2467 (2008).
